# Existence of benefit finding and posttraumatic growth in people treated for head and neck cancer: a systematic review

**DOI:** 10.7717/peerj.256

**Published:** 2014-02-11

**Authors:** Sam Harding, Fatimeh Sanipour, Timothy Moss

**Affiliations:** 1 Department of Psychology, University of the West of England, Bristol, United Kingdom; 2 Centre for Appearance Research, University of the West of England, Bristol, United Kingdom

**Keywords:** Posttraumatic growth, Benefit finding, Head and neck, Cancer, Silver lining questionnaire, Posttraumatic growth inventory, Quality of life, Systematic review

## Abstract

**Background.** The impact of head and neck cancer (HNC) in long-term survivors differs widely among individuals, and a significant number of them suffer from the negative effects of disease, whereas others report significant positive effect. This systematic review investigated the evidence the implications of treatment for HNC and subsequent development of Benefit Finding (BF) or Posttraumatic Growth (PTG).

**Purpose.** To understand how differing medical, psychological and social characteristics of HNC may lead to BF/PTG and subsequently inform post-treatment interventions to encourage positive outcomes.

**Method.** In February 2012, five databases including Pubmed, and Psych Info, were searched, for peer-reviewed English-language publications. Search strings included key words pertaining to HNC, BF, and PTG. One thousand three hundred and sixty three publications were identified, reviewed, and reduced following Cochrane guidelines and inclusion/exclusion criteria specified by a group of maxillofacial consultants and psychologists. Publications were then quality assessed using the CASP Cohort Critical Appraisal tool.

**Findings.** Five manuscripts met the search and selection criteria, and were sourced for review. All studies were identified as being level IIb evidence which is a medium level of quality. The majority of studies investigated benefit finding (80%) and were split between recruiting participant via cancer clinics and postal survey. They focused on the medical, psychological and social characteristics of the patient following completion of treatment for HNC.

**Conclusion.** Demographic factors across the papers showed similar patterns of relationships across BF and PTG; that higher education/qualification and cohabitation/marriage are associated with increased BF/PTG. Similarly, overlap with disease characteristics and psychosocial factors where hope and optimism were both positively correlated with increased reported BF/PTG.

## Introduction

A great deal of evidence has accumulated over the past thirty years for the negative sequelae of trauma. Traumatic events can include a range of experiences including health threats. The literature on coping with health difficulties has documented a variety of negative consequences, including; depression (e.g., Moyer & Salovey, 1996; Timberlake *et al.*, 1997, posttraumatic stress disorder (PTSD) (e.g., Alter *et al.*, 1996; Andrykowski *et al.*, 1998, and adjustment difficulties (e.g., Schulz *et al.*, 1995). These models have tended to work towards a clinical diagnosis for which treatment may be prescribed.

By contrast, models of positive illness recovery have been informed by a range of more general theories of life change (Horowitz, 1986; Park & Ai, 2006; Paton, 2006). These have tried to understand the mechanisms that may underpin the positive sequelae of health-related trauma. Since these models are not working towards a diagnosis for prescription, there has been no imperative to coalesce around a common agreed understanding against which a diagnosis can be made.

Morse (1997) conceptualises coping with life-threatening illness as incorporating five distinct stages. The first stage is uncertainty or vigilance, during which patients suspect a condition and attempt to maintain emotional control whilst trying to understand their condition and its severity. The second stage is disruption, a time when individuals realise that they are affected by what they perceived to be a serious disease and may experience high levels of stress. In the third stage, striving for recovery, individuals may try to gain control over their illness with the help of personal and environmental resources. The fourth stage is striving to restore one’s self and making sense of altered reality. The fifth and final stage is learning to live with the altered self, in which patients attain a new equilibrium as a result of accepting the illness and its consequences. In chronic illness, a return to a prior state of health may not be a realistic outcome. This and subsequent models suggest that it is the time of diagnosis, and the disruption stage, especially when this involves news of a life-threatening illness, that patients are the most likely to experience trauma (Morse & Johnson, 1991). This is also the stage during which individuals are most likely to confront existential issues posed by the diagnosis (Doka, 2008).

Brennan (2001) proposes that social cognitive transition (SCT) model builds on previous theories of coping, traumatic stress, social-cognition and cognitive theories of emotion. This theory hinges on the central components of the cognitive models of PTSD, except it allows for both positive and negative psychological outcomes after a trauma. Brennan (2001) proposes that all individuals have mental models of the world, made up of assumptions. As an individual interacts with their world these assumptions are either confirmed or disconfirmed by experience. If we consider Leventhal’s model of Self-regulation (Leventhal, Nerenz & Steele, 1984), then his stimulus is a disruption or challenge to the Assumptive World. The arising representations map to an expectation, and the coping behaviours to new experiences. The subsequent outcomes either confirm or disconfirm the mental model of the Assumptive World. In this way, Brennan’s medical model encompasses Leventhal’s broader psychosocial framework and provides an account for the diverse psychosocial outcomes experienced by cancer patients.

This model would propose that PTSD is the negative result of an extremely troubling event that is highly incongruent with the individual’s assumptions about the world. Brennan (2001) indicates that denial and avoidance are the first responses of a traumatised individual, which create more stress and potentially lead to the development of new assumptions about the world, assumptions that may be dysfunctional and lead to heightened levels of distress or PTSD. However, avoidance and denial can also serve a positive roll by diluting “the absorption of ‘traumatic’ information” (Brennan & Moynihan, 2004, p. 9). Conversely, Brennan & Moynihan (2004) proposes that an adaptive response to traumatic experiences requires worry. It is hypothesised that worry is a part of the cognitive attempt to anticipate and prepare for future threat (Brennan & Moynihan, 2004; Eysenck, 1992). By imagining and confronting worst case scenarios, by “decatastrophising” them, the individual can appraise the realistic nature of the event. Brennan & Moynihan (2004) proposes that positive outcomes from traumatic experiences can then occur, as unrealistic goals or outcomes are discarded and implicit long-standing life goals become clear and distinct.

Benefit finding (BF) and posttraumatic growth (PTG) describe similar outcomes following adversity, yet there are clear differences. Both describe a positive outcome with BF being described as the acquisition of benefit from adversity (Collins, Taylor & Skokan, 1990; Tennen & Affleck, 2002) and PTG growth being the success with which individuals coping with the aftermath of trauma reconstruct or strengthen their perceptions of self, others and the meaning of events (Tedeschi & Calhoun, 1996). Examples of BF finding include a positive change in relationships, a greater appreciation of life and a change in life priorities. PTG is also described as ‘the experience of significant positive change arising from the struggle with a major life crisis’, with examples of increased sense of personal strength, changed priorities and richer existential and spiritual life being cited in the literature (Calhoun *et al.*, 2000).

Despite these similarities, there is emerging evidence that there are critical differences, for example, Sears, Stanton & Danoff-Burg (2003) showed that BF was predicted by personal characteristics (i.e., education, optimism, and hope), but PTG was not. Benefit finding may start immediately after diagnosis and results from challenges to the individual’s cognitive representations; that is, they have the same personal representations, but have positive ways of coping. By contrast, PTG is a re-assembly of the assumptive world in a new way following trauma and develops as a result of the rumination and restructuring of the self/world relationship, that occurs in the weeks, months, and even years following trauma and is focussed on changes in one’s capacity to deal with adverse events (Calhoun & Tedeschi, 1998). So PTG results from challenges to deeper cognitive representations than BF and result in changed ‘rules for living’ and ‘core schema’, whereas BF may be more superficial and transient in nature. This difference may also lead one to expect more PTG growth with increasing time post-trauma, because more time is available for cognitive processing (Sears, Stanton & Danoff-Burg, 2003).

However, this hypothesis has yet to be tested and given that PTG has no diagnostic period of onset, unlike PTSD (American Psychiatric Association, 2013), this systematic review has aggregated BF and PTG and will search for both of these concepts and words/phrase used synonymously such as ‘stress-related growth’ and ‘existential growth’. The authors will refer to these concepts throughout the remainder to this manuscript as BF/PTG unless making specific reference to information from research where one theoretical perspective has been purposely selected.

Recent studies have provided evidence that these positive processes also take place in chronically ill patients, including individuals suffering from cancer (Affleck & Tennen, 1996; Carver & Antoni, 2004; Petrie *et al.*, 1999; Schulz & Mohamed, 2004; Sears, Stanton & Danoff-Burg, 2003; Tomich & Helgeson, 2004). The bulk of this research has been undertaken on females with breast cancer (Carver & Antoni, 2004; Petrie *et al.*, 1999; Sears, Stanton & Danoff-Burg, 2003; Tomich & Helgeson, 2004). There have also been some general cancer review papers published, but none which have focused on people with head and neck cancer (Stanton, Bower, & Low, 2006; Sumalla, Ochoa, & Blanco, 2009). In the United Kingdom 125.9 females in every 100,000 will suffer from breast cancer and 1.0 males. For oral cancer the figures are 5.5 and 12.4 respectively (Cancer Research UK, 2013). Additionally Cancer Research UK (2013) statistics indicate that people with oral cancer are older at diagnosis than those with breast cancer. These two factors combined with the location of the tumour may impact the development of BF/PTG, and it is for this reason that a systematic review of this cancer site is needed.

This systematic review investigates the literature on BF/PTG in the patients treated for cancer in the region of the Head and Neck (HNC). The aim is to collate the current quantitative data to understand how differing medical, psychological and social characteristics of HNC may lead to BF/PTG and subsequently may inform diagnosis and future post-treatment interventions to encourage sustained positive outcomes.

## Methods

The review strategy was adapted from the Cochrane Collaboration systematic review methodology and uses a narrative synthesis (The Cochrane Collaboration, 1999) and guidance from Petticrew & Roberts (2006).

### Identification of selection criteria

The Booth & Fry-Smith (2004) PICO model (population, intervention, comparison, outcome) guided the development of the search strategy.

The ‘Population’ of interest was defined as adults (>18 years) of either sex with HNC. Children and adolescents can develop HNC, but due to high relevance of developmental stage, and cognitive maturity they are excluded from the review. Terminal patients and those with recurrent metastatic disease on entry to the study were excluded, as they would currently be experiencing significant on-going challenging and potentially traumatic experiences.

This systematic review is not investigating an ‘Intervention’ in the sense of ‘Cognitive Behavioural Therapy’, as an example. The interventions of interest that may affect outcome is the treatment for the malignant tumour, i.e., surgery, radiotherapy, chemotherapy and any combination of these treatments, or specifically named variations such as photodynamic therapy. In relation to ‘comparisons’, no limitations were put on the search strategy. However it was noted that comparison may be possible by simply comparing intervention groups, cancer sites (Table 1) or measure pre and post intervention.

**Table 1:** ICD10 codes related to cancer sites and incidence.

Cancer site	ICD10 code	Number of registrations 2000	Incidence: crude rate per 100,000, 2000	
			Men	Women
Mouth, lip & oral cavity	C00-06	2329	5.9	3.7
Salivary glands	C07-8	422	1	0.8
Pharynx	C09-14	1339	4	1.6
Nasal cavity, ear & sinuses	C30-31	352	0.8	0.6
Larynx	C32	1903	6.6	1.3
Thyroid	C73	1131	1.3	3.3

When considering the relevance of ‘outcome’ measures to the development of the search strategy, this review focused purely on quantitative studies. The studies must include ‘paper and pencil’ or ‘computer based’ psychometrically sound measures of BF and/or PTG. This will allow comparison of statistical analysis of the relationship between BF/PTG and categorical medical and social variables, as well as other psychological characteristics collected via validated measures. Data collected via studies reporting qualitative data only were excluded.

### Search strategy

**Figure 1:**
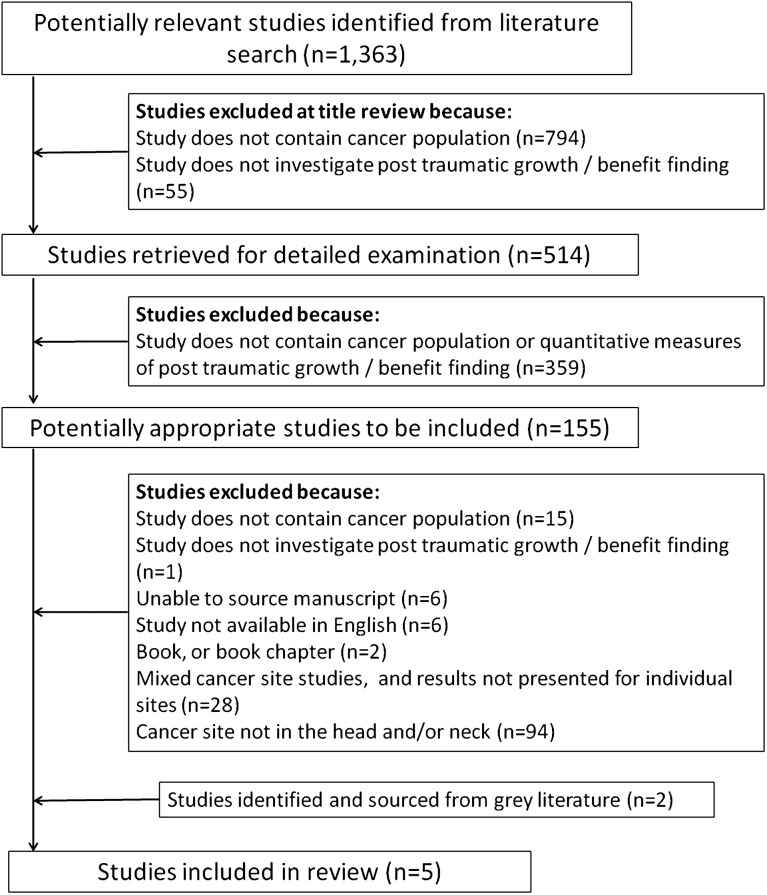
PRISMA flowchart.

The search strategy was designed in consultation with a senior librarian and the search terms following a review of the literature and discussion with a Maxillofacial Consultant (Supplemental Information A). A combination of ‘free text’ terms with Boolean operators and truncations were used. Five separate searches were conducted in electronic databases; Pubmed, Psych Info (CSA), Psyc Articles (CSA), OVID Medline, and PILOTS (Published International Literature on Traumatic Stress), to identify appropriate studies in articles published from the earliest entries of any of the databases until February 2012. No limits were placed on the electronic search in relation to age range of participants studied or language of publication. The PRISMA checklist was followed and a flow chart (Fig. 1) details the process of article selection.

The citations retrieved from each database were exported to ‘Reference Manager 11’ bibliographic management software (Thomson ResearchSoft, 2000). Duplicates were removed, and article screened for relevance, removing animal studies and medical and psychological studies which had been retrieved as they contained one or more of the search terms, e.g., Squamous Cell or Benefit (Supplemental Information B). To this point in the review process no limits or restrictions had been placed on ‘cancer site’ while searching the electronic databases or retrieved articles. This enabled papers reporting on multiple cancer sites to be identified and integrated for patterns between tumour locations. Supplemental Information B provides the list of search terms used to identify appropriate tumour locations within the head and neck region. We did not limit the search to include or exclude any type of intervention within this participant cohort. In this review, an intervention would be the type of cancer treatment they received. Cancer location and treatment were specific factors that were identified as potential confounders/variables within the selected papers, but this did not require additional terminology to be added to the research strings or strategies. The 514 abstracts of the remaining articles related to BF, PTG and/or cancer were screened by SH and twenty percent randomly sampled were reviewed by TM and FS.

Guidelines, dissertations and theses greater than 5 years old, handbooks, commentaries, review articles, expert opinions and case reports, as well as trials with fewer than ten participants were excluded, as were qualitative studies. Disagreement between the review authors was resolved by consensus through discussion. This identified ‘potentially relevant articles’ (*n* = 155) and these were obtained and appraised critically.

Three articles (Harrington, McGurk & Llewellyn, 2008; Ho *et al.*, 2011; Llewellyn *et al.*, 2011) were identified from this search strategy. After completing the literature search, references from these articles, review articles, thesis and books were examined to identify additional grey literature and the author (SH) contacted researchers identified. Two projects were identified, but no responses were received when the authors were contacted. Two of the authors of this Systematic Review (SH & TM), have two manuscripts in preparation for submission and these were included in this review as grey literature (S Harding & T Moss, 2013a, unpublished data; S Harding, T Moss, 2013b, unpublished data).

The five identified manuscripts were summarised separately, including a description of the study design, sample size, measurement, and time since diagnosis or treatment of HNC, and are presented in Tables 2 and 3.

**Table 2:** Study descriptors.

Study	Author(s)	Aim of the study	Study design	Study measures	Demographic factors	Medical factors	Time of measurement
1	Harrington, McGurk & Llewellyn (2008)	(1) to determine the extent to which patient treated for HNC experience positive consequences of their illness, (2) to identify factors associated with benefit finding among this patient group	Cross-sectional postal survey	Benefit finding scale (BFS), Hospital Anxiety and Distress Scale (HADS), Life Orientation Test-Revised (LOT-R), Brief COPE	Age, Gender, Ethnicity, Education, Employment, Marital status	Type of treatment, time since last treatment, diagnosis of further illness since treatment, site, type of cancer and stage of cancer	0–6mths = 1, 6–12mths = 3, 13–24mths = 7, 25–47mths = 20, 48–72mths = 19, 73–121mths = 26
2	Llewellyn *et al.* (2011)	(1) to determine the extent to which patient treated for HNC experience positive consequences of their illness, (2) to establish the relationship between BF, other patient-reported outcomes and predictive factors such as coping strategy and level of optimism	Repeated measures prospective study using self-completion questionnaires	Benefit finding scale (BFS), Hospital Anxiety and Distress Scale (HADS), Life Orientation Test (LOT-R), Brief COPE, Medical Outcomes Short Form 12 (SF-12), Two-item measure derived from The European Organization for Research and Treatment (EORTC) of Cancer Quality of Life Questionnaire (QLQ-C30)	Age, Gender, Ethnicity, Education, Employment, Marital status	Type of treatment, site and stage of cancer	T1 = Between diagnosis and start of treatment, T2 = 6 months after completion of treatment
3	Ho *et al.* (2011)	Investigate if PTG occurs in oral cancer patients and if hope and optimism shows significant positive correlation with PTG	Cross-sectional postal survey	Chinese Posttraumatic Growth Inventory (PTGI), Hope scale (HS), Life Orientation Test - Revised (LOT-R)	Age, Gender, Religion, Education level, income	Time since diagnosis, stage of disease, and treatment type	Mean time was 3.6yrs (SD 0.34)
4	S Harding & T Moss, (2013a, unpublished data)	Investigate the relationship between BF, demographical, biomedical and HRQoL following the treatment for HNC	Cross-sectional postal survey	Silver Lining Questionnaire (SLQ), University of Washington Head and Neck Caner Quality of Life (UoW), Medical Outcomes Short Form 12 (SF-12)	Age at diagnosis, Age at time of completing questionnaire, Gender, Ethnicity, Index of Multiple Deprivation, Occupation, Family Status	Tumour site, Stage of disease, Location of tumour, Treatment	Mean time from completing treatment to completing questionnaires 27.30mths (Range 3–76; SD 21.8)
5	S Harding & T Moss, (2013b, unpublished data)	Investigate the longitudinal relationship between BF, demographical, biomedical and HRQoL following the treatment for HNC	Repeated measures prospective cross-sectional study using self-completion questionnaires	Silver Lining Questionnaire (SLQ), University of Washington Head and Neck Caner Quality of Life (UoW), Medical Outcomes Short Form 12 (SF-12)	Age at diagnosis, Age at time of completing questionnaire, Gender, Ethnicity, Index of Multiple Deprivation, Occupation, Family Status	Tumour site, Stage of disease, Location of tumour, Treatment	

**Table 3:** Participants and variables.

Study	Author(s)	Participants (gender, age)	Time of measurement	Non-respondents/dropouts	Exclusion criteria	Cancer site	Cancer staging	Cancer treatments	Time since completion of treatment
1	Harrington, McGurk & Llewellyn (2008)	*N* =76 (55% response rate; 37 Male, 39 Female; Mean Age 66.9, SD 12.6, Range 32–97; 71 White)	0–6mths = 1, 6–12mths = 3, 13–24mths = 7, 25–47mths = 20, 48–72mths = 19, 73–121mths = 26	Significant difference between gender in responders and non-responders (more females responding)	Under 18 years of age. Having palliative treatment. Recurrent diagnosis, metastatic disease in other parts of the body (excluding neck nodes), a diagnosis of lymphoma, mental to cognitive impairments or insufficient understanding of English.	Not stated	Stage 1–2 - *N* = 53, Stage 3–4 - *N* = 23	Surgery only - *N* = 35, Radiotherapy only - *N* = 10, Surgery and Radiotherapy - *N* = 30, Surgery, radiotherapy and chemotherapy - N = 1	0–6mths = 1, 6–12mths = 3, 13-24mths = 7, 25–47mths = 20, 48–72mths = 19, 73–121mths = 26
2	Llewellyn *et al.* (2011)	T1. *N* = 103 (73 Males, 30 Females; Mean Age 63, SD 13.9, Range 23–91; 93 White). T2. *N* = 68 (Gender, Age, Ethnicity data provided)	T1 = Between diagnosis and start of treatment, T2 = 6 months after completion of treatment	There were no significant differences between patients included and not included with respect to gender, stage of cancer. 35 people did not complete the second time point. No information is given about they compared at T1	Under 18 years of age. Having palliative treatment. Recurrent diagnosis, metastatic disease in other parts of the body (excluding neck nodes), a diagnosis of lymphoma, mental to cognitive impairments or insufficient understanding of English.	Oral Cavity - *N* = 68, Pharynx - *N* = 8, Larynx - *N* = 19, Other - *N* = 8	Stage 1 - *N* = 34, Stage 2 - *N* = 25, Stage 3 - *N* = 23, Stage 4 - *N* = 17, Missing data - *N* = 4	Surgery only - *N* = 36, Radiotherapy only - *N* = 25, Chemotherapy only - *N* = 3, Surgery and Radiotherapy - *N* = 17, Radiotherapy and chemotherapy - *N* = 13, Surgery, radiotherapy and chemotherapy - *N* = 9	Six months at T2
3	Ho *et al.* (2011)	*N* =50 (21 Male, 29 Female; Mean Age 60, SD 13.06)	Mean time was 3.6yrs (SD 0.34)	No information is reported	Non-native Cantonese speakers, less than 6mths post treatment completion, recurrence	Oral Cavity, Oropharynx, gingival, floor of mouth, tongue, salivary glands, buccal mucosa, palate. Numbers at each site not stated.	Stage 1–2 - *N* = 41, Stage 3–4 - *N* = 5, Missing information - N = 4	Surgery only - *N* = 34, Surgery and Radiotherapy - *N* = 16	Mean time was 3.6yrs (SD 0.34)
4	S. Harding & T. Moss, (2013a, unpublished data)	*N* =164 (55% response rate; 108 Male, 56 Female; Mean Age 67.0yrs, SD 12.5)	Mean time from completing treatment to completing questionnaires 27.30mths (Range 3–76; SD 21.8)	One difference was found between responders and non-responders with a greater number of people from less deprived areas returning questionnaires	Less than 3mths post treatment completion, recurrence	Oral Cavity - *N* = 68, Oropharynx - *N* = 43, Hypo pharynx - N = 17, Larynx - *N* = 36	Stage Tis - *N* = 2, Stage 1 - *N* = 39, Stage 2 - *N* = 37, Stage 3 - *N* = 30, Stage 4 - *N* = 55, Missing data - *N* = 1	Surgery only - *N* = 52, Radiotherapy only - *N* = 35, Chemotherapy only - *N* = 1, Surgery and Radiotherapy - *N* = 35, Surgery and chemotherapy - *N* = 3, Radiotherapy and chemotherapy - *N* = 24, Surgery, radiotherapy and chemotherapy - *N* = 14	Mean time from completing treatment to completing questionnaires 27.30mths (Range 3-76; SD 21.8)
5	S. Harding & T. Moss, (2013b, unpublished data)	*N* =163 (55% response rate; 105 Male, 58 Female; Mean Age 68.6yrs, SD 11.2)	Mean time from completing treatment to completing questionnaires TP1 = 32.2mths (Range 3-113; SD 27.8), TP2 = 45.1mths (Range 15–125; SD 28.1)		Less than 3mths post treatment completion, recurrence	Oral Cavity - *N* = 75, Oropharynx - *N* = 33, Hypo pharynx - N = 24, Larynx - *N* = 31	Stage Tis - *N* = 2, Stage 1 - *N* = 38, Stage 2 - *N* = 35, Stage 3 - *N* = 32, Stage 4 - *N* = 47, Missing data - *N* = 9	Surgery only – *N* = 48, Radiotherapy only - *N* = 35, Chemotherapy only - *N* = 0, Surgery and Radiotherapy - *N* = 44, Surgery and chemotherapy - *N* = 2, Radiotherapy and chemotherapy - N = 17, Surgery, radiotherapy and chemotherapy - *N* = 17	Mean time from completing treatment to completing questionnaires TP1 = 32.2mths (Range 3–113; SD 27.8), TP2 = 45.1mths (Range 15–125; SD 45.1)

One of the five identified papers did not provide sufficient data to extract as part of this review. The authors of that article were approached and subsequently provided an additional publication that enabled a fuller understanding of their data and greater comparison with other published work (Horney *et al.*, 2011).

### Quality assessment

This review has identified a very limited number of studies; it is therefore insufficient to limit the assessment of papers to those with the ‘best’ methodology. The studies identified in this review all represented ‘level IIb’ evidence (Supplemental Information C; National Institute for Clinical Excellence, 2004), or those at a medium level of quality, where high levels would refer to studies in the top of the hierarchy of evidence (e.g., systematic reviews, randomised controlled trials), and ‘low’ refers to those near the bottom of the hierarchy (case series, case reports, expert opinion). Given this assessment of quality, the remaining assessment of quality reflects variation within that small banding.

Quality was assessed using the Critical Appraisal Skills Programme (CASP) Cohort Study appraisal tools (Critical Appraisal Skills Programme, 2011). This tool provides a 12 point check list of study validity, risk of bias in recruitment, exposure, outcome measurement, confounding factors, reporting of results and the transferability of findings (maximum score of 12). The key questions from CASP were taken as a template for the quality appraisal (Supplemental Information D). The appraisal questions were answered with ‘yes’, ‘can’t tell’ and ‘no’. Where ‘yes’ was used, the study was felt to fill the criteria for that question. Where ‘can’t tell’ was used, the study was considered to meet some of the criteria for the question, but not others. Where ‘no’ was used, the study was considered to explicitly not meet the criteria for the question. CASP does not provide cut-offs for quality levels, however no studies were ruled out on the basis of the quality appraisal since quality levels were similar between studies.

All identified manuscripts were checked for quality against the appraisal tool independently by SH and FS and confirmed by TM. Consensus was immediate between the reviewers. Each of the scales used within the studies were also assessed and reported (Supplemental Information E). Upon reviewing the studies’ data collection tools and statistical analysis it became apparent that there was too great a variation between them and thus it was not appropriate to conduct additional analysis such as a meta-analysis using the reported findings.

## Results

### Quality Assessment Findings

The fashion in which data is collected may affect the results. Two of the included studies collected the data during patients’ clinic visits (Ho *et al.*, 2011; Llewellyn *et al.*, 2011). This may have increased the potential sample size, but it may also have caused the respondents to report positive outcomes due to feelings of appreciation for medical treatment, or as a means of thanking the clinical team for treatment. The other three studies posted the measures to the participants, which is less likely to elicit socially desirable responses (S. Harding & T. Moss, 2012a, unpublished data; S. Harding & T. Moss, 2012b, unpublished data; Harrington, McGurk & Llewellyn, 2008). Postal surveys can result in a low return rate, although those reviewed here received 53–55% (respectively S. Harding & T. Moss, 2013a, unpublished data; Harrington, McGurk & Llewellyn, 2008) and can be argued to be reasonable. A separate consideration is that they may be biased through participants self-selecting and subsequently call into the question the generalisability of the findings.

All the studies included in this review were quantitative in nature, and used previously constructed measures (Supplemental Information E). Measures such as the Medical Outcomes Short Form 12 (SF-12) have normative date that allows findings to be compared with general population (S. Harding & T. Moss, 2013a, unpublished data; S. Harding & T. Moss, 2013b, unpublished data; Llewellyn *et al.*, 2011). Other measures have only been used in other disease populations, such as hospital anxiety and depression scale (Harrington, McGurk & Llewellyn, 2008; Llewellyn *et al.*, 2011). An exception to this was one of the measures used in Llewellyn *et al.* (2011). In this study, two items were derived from the EORTC QLQ-C30, which were used to assess cancer specific global Quality of Life/health status.

In medical population studies the confounding factors such as stage or exact location of tumour may be predictive factors and it is therefore important to ensure that these are appropriate selected and analysed (Bellizzi & Blank, 2006; Brunet *et al.*, 2010; Gallagher-Ross, 2012). Similar factors were used across all studies included in this review and were sourced from individual patient records and electronic hospital databases. It was therefore believed that all these would be accurate and allow for non-responder comparisons reported by Harding & Moss (2013a, unpublished data) and Llewellyn *et al.* (2011) to be authentic.

Overall the quality of the five reviewed articles are of a medium level. They represent a small total population of 343 people with HNC completing quantitative measure or sub-scales of measures. Insufficient data is presented from the combined sample size, or from anyone measure to allow for meta-analysis of the impact of treatment methodology, cancer site, or staging. Additionally the two papers by Harding and Moss (2013a, unpublished data; 2013b, unpublished data) have not undergone peer review and therefore need to be considered cautiously.

### Demographic factors related to BF in HNC patients

The reviewed BF studies each collected a large number of demographic variables hypothesised as predictive or correlated with BF. Harrington, McGurk & Llewellyn (2008) undertook the first investigation into BF in the HNC patient population; however, they did not find any demographic variables correlating with BF. The subsequent work from the same research group (Llewellyn *et al.*, 2011) found that there was a positive association between BF and being married or cohabiting and living alone, as well as with higher educational qualifications. Harding and Moss (2013a, unpublished data) added to this by finding that the younger the patient at time of diagnosis the greater the associated BF. Harding & Moss (2013b, unpublished data) longitudinal study further supported this relationship with the age at time of diagnosis being correlated with reported BF over both time periods.

### Demographic factors related to PTG in HNC patients

Only one paper was identified as having specifically investigated PTG (Ho *et al.*, 2011). Age and time since diagnosis did not show any significant relationship. Nor was there any significant difference in relation to religion or gender. Economic status showed significant relationship with PTG, with patients form the higher income reporting higher posttraumatic growth inventory (PTGI) scores. Education level, however, did not show any significant effect on PTG. As with BF, marital status showed significant association with PTG. Comparing married patients and patients not in a relationship showed that married patients reported higher scores on PTGI. Analysis showed that married patients reported higher total hope scores than their unmarried counterparts.

### Relation of BF to disease characteristic and psychosocial factors in HNC patients

Harrington, McGurk & Llewellyn (2008) found that dispositional optimism and positive reframing could account for 23% of variance in BF and additionally that higher levels of religious coping was correlated with greater BF. Harrington, McGurk & Llewellyn (2008) did not find any relationship between BF and Anxiety, Depression, Time since treatment, Treatment, Stage of Cancer or diagnosis of further illness and this pattern was reinforced by the findings of Llewellyn *et al.* (2011). Llewellyn *et al.* (2011) supported the finding related to dispositional optimism and positive reframing, but also found that an increased use of emotional support and a decrease in self-blame positively affect BF. This combination of factors was found to account of 39% of BF variance. Harding and Moss (2013a, unpublished data) investigates subscales of BF; (1) ‘Perceived changes in self’ (2) ‘Changes in interpersonal relationships’ and (3) ‘Changes in spirituality or the philosophy of life’ using the Silver Lining Questionnaire (SLQ-Sp). They found that the less pain the patient is experiencing the more PTG they report across all three domains. Other significant correlations found within the SLQ showed that when participants did not suffer with movement restrictions, they reported greater changes in SLQ. Greater SLQ was experienced by people whose mood ‘is excellent and unaffected by their cancer and also those who are ‘as active as ‘they’ have ever been’.

Llewellyn *et al.* (2011) found that an increase in emotional growth was negatively related to the mental component summary (MCS) score. This indicates that higher levels of emotional growth are associated with poorer mental health related Quality of Life. This pattern is supported by Harding and Moss (2013a, unpublished data) who also found that MCS in HNC treated patients was significantly worse than the normative population. However, Harding & Moss (2013b, unpublished data) failed to find this pattern with the MCS longitudinally, in fact the ‘mood’ subscale of the University of Washington (UoW) scale suggested that the less the individuals mood is disturbed by their cancer the more BF they report. The same pattern was found with the ‘activity’ and ‘recreation’ sub scale of UoW.

### Relation of PTG to disease characteristic and psychosocial factors in HNC patients

Ho *et al.* (2011) found that patients with more advanced cancer stages III and IV reported lower levels of PTG, but that different treatment modalities did not significantly influence PTG. Regarding the hope scale, the life orientation test-revised, and the PTGI correlation showed a positive relationship between hope and optimism. Both, hope and optimism are positively correlated to PTGI.

Results of regression analyses comparing hope and optimism in relation to PTG found that hope and optimism contributed to a 25% variance of PTG. However, only hope was a significant individual indicator of PTG.

## Discussion

The primary aim of this review was to evaluate the evidence which assesses the potential relationship between BF/PTG and medical, social and psychological variables as measured by validated scales people who have suffered from HNC. Posttraumatic growth is a rapidly developing field of research (Larick & Graf, 2012; Kunst, 2012; Li *et al.*, 2012), but new and developing in the particular patient cohort (HNC) selected for this systematic review.

Because it has been suggested that BF and PTG are conceptually different constructs the authors looked at the BF manuscripts separately (S. Harding & T. Moss, 2013a, unpublished data, S. Harding & T. Moss, 2013b, unpublished data, Harrington, McGurk & Llewellyn, 2008, Llewellyn *et al.*, 2011) to the PTG manuscript (Ho *et al.*, 2011). However, the demographic factors across the papers show a similar pattern of relationships across the constructs; that higher education/qualification and cohabitation/marriage are both associated with reported increased BF/PTG. Similarly, there is overlap with BF/PTG in HNC patients with disease characteristics and psychosocial factors where hope and optimism are both positively correlated with increased reported BF/PTG. Very few associations were observed with any of the HNC biomedical or disease factors and BF/PTG.

### Methodological limitations of this paper

Although clear systematic criteria were used for search and inclusion strategies, it is possible that a number of biases may enter into the process by way of variations in definitions (e.g., of the BF and/or PTG construct), and in general by the narrow inclusion criteria. For example, by including quantitative empirical studies only, the possibility of deriving a fuller understanding of the mechanisms underlying any relationships between PTG and HNC remains limited. However, for the purposes of this review, we focused on the given inclusion criteria in order to carefully accumulate the literature on PTG and HNC with a view to developing a picture of the current status of empirical findings.

The limited number of the studies available for review makes it difficult to draw firm conclusions and develop hypotheses about how differing characteristics and conditions may lead to BF/PTG, and how they may inform future post-treatment interventions to encourage positive psychosocial outcomes. The inclusion of unpublished data is always a point for specific consideration, however, in this review the unpublished data was provided in addition to published data on BF. The unpublished data was specifically considering the phenomenon in question and was not given undue weight in analysis. For this reason it has been included, but rightly noted as a limitation.

In this review the primary author (SH) reviewed and evaluated all the retrieved abstracts and selected papers with twenty percent checks undertaken by co-authors. In addition the two manuscripts by the authors of this review (SH &TM), were reviewed by independent peer reviewers. This procedure has previously been validated by the Agency for Healthcare Research and Quality (Hartling *et al.*, 2012).

The results are important contributions to the limited information available on both PTG and BF in HNC survivors. The overlapping patterns observed between the PTG and BF studies suggest that simultaneous study of the two concepts would provide insight into the conceptual distinction. Mols *et al.* (2009) point out that the impact of cancer in long-term survivors differs widely among individuals, and a significant number of them suffer from the negative effects of disease, where as others report significant positive effect. This dichotomy of concepts should be familiar to all allied health care professionals, but they should be mindful of the potential consequences of trying to impose expectations of patients (Bellizzi & Blank, 2006). In relation to developing an intervention it is important to identity patient characteristics (e.g., optimism, returning to work, life satisfaction) that can be manipulated in order to promote BF and PTG. If these characteristics are known, theory driven interventions may be developed to alter them and reduce risk of negative effects and increase positive ones.

### Limitations of reviewed studies

Results stemming from these studies are valuable; however, some limitations and methodological considerations should be noted. First, three of the five studies were cross-sectional in design, thus they provided the authors with limited knowledge about the temporal course of the conditions and the direction of causality between them and the related factors. It has been suggested by some models that it is the time of diagnosis that can be the onset stimulus (Doka, 2008; Morse, 1997), but no firm evidence has been forthcoming. This makes it difficult to draw conclusions from the findings of Llewellyn *et al.* (2011) because it may be that simply diagnosing cancer is significant enough to start patients BF which is sustained through to six months post treatment, therefore explaining the lack of difference found between the two time points. Additionally, it is not obvious whether time since diagnosis has an effect on the development of BF/PTG; only a longitudinal study would allow researchers to draw firmer conclusions about the role each suggested factor plays in the onset of PTG.

Moreover, because four studies were asking the patients retrospective questions, the possibility of distortion of results from recall bias is increased. It is possible that a patient cannot remember exactly how much support they received, for example, lifts to the hospital, people waiting for them during treatment, collection of medication from pharmacists, picking up shopping supplies. The reviewed studies relied on self-reported measures, which might be susceptible to reporting bias, according to the participant’s mood or opinion or even as a result of post hoc bolstering (Zoellner & Maercker, 2006), thus possibly enhancing the likelihood of distorted results and the requirement for sufficiently large sample populations to account for the variability that this may introduce.

The measures used (Supplemental Information E), though being psychometrically validated, also have some restrictions. Llewellyn *et al.* (2011) used two items from the EORTC QLQ-C30, which leads to questionable interpretation of the data, as the items have been de-contextualised and therefore no longer actually measure what they claim. The Benefit Finding Scale incorporates both positively and negatively phrased items into questionnaires. The purpose for this is to counter the effects of social desirability and acquiescence (Nunnally, 1978). However statistical analysis of this scale has found that respondents answered the negatively phrased items differently to the positively phrased items, affecting score validity. Schriesheim & Eisenbach (1995) have subsequently identified three important assumptions underlying the use of balanced scales. First, acquiescence is a serious threat to the validity of score interpretation. Second, the negatively worded and positively worded items are bipolar statements within the same construct. Third, negatively worded items can be used without major adverse side-effects on the psychometric properties of the instrument. However, this may only become apparent when items are subjected to factor analysis in future work.

Another methodological limitation is that statistical analyses of studies searched only for linear relationships between BF/PTG and relevant variables. Some investigators have found curvilinear relationships between PTG and psychosocial variables might be present, for example between level of distress and BF (Lechner *et al.*, 2006) and mental health and well-being (Seery, 2011). An additional advance that could be made would be to use a control group of healthy participants to determine whether the positive changes reported stemmed from the trauma, or were simply the normal effect of time passing (e.g., aging), which affects individuals in multiple ways.

It is also worthwhile discussing some limitations regarding the samples examined in the included studies. The three published studies recruited (or retained for analysis) small sample sizes of fewer than 100 participants (Harrington, McGurk & Llewellyn, 2008; Ho *et al.*, 2011; Llewellyn *et al.*, 2011). It is recommended that for each variable being measured at least 10 participants be recruited (Pallant, 2010) and that a more conservative level of significance (e.g., *P* ≤ 0.001 instead of *P* ≤ 0.05) be required before conclusions can be drawn. The limitation with the small sample size studies is that the large number of variables being assessed may introduce Type I errors. Three of the five studies followed the sample size guidance (S. Harding & T. Moss, 2013a, unpublished data; S. Harding & T. Moss, 2013b, unpublished data; Llewellyn *et al.*, 2011. By contrast, the Harrington, McGurk & Llewellyn (2008) study may have failed to find statistically significant differences as the analysis of 76 respondents is likely to under-powered; with 15 variables the Wilson Van Voorhis & Morgan (2013) guidelines suggest a minimum of 105 respondents for correlation and 300 for factor analysis.

Another issue is that all the studies relied on convenience samples of volunteers in which minorities were under-represented, and relatively homogeneous samples were recruited, which challenges the generalisability of the findings. Additionally there were differences in relation to socio-economic status and ethnicity across people that responded and those that did not respond to the postal surveys. The lower recruitment rates of postal surveys to clinic surveys may be due to perceived pressure felt by people at clinic appointments. It is possible that these different methodologies affect how the questionnaires are completed and consequently the findings. However due to the small sample sizes and limited number of studies, no directional hypothesis can be made.

### Future Directions

As CASP (Critical Appraisal Skills Programme, 2011) notes ‘one observational study rarely provides sufficiently robust evidence to recommend changes to clinical practice or within health policy decision making’. The present review offers a summary of the limited work on BF and PTG research in relation to HNC treatment.

Future research might usefully focus on providing a review of qualitative studies in this area in order to generate further hypotheses reflecting the possible association between BF, PTG and HNC. Within the current review careful attempts were made to complement this method with objective criteria (e.g., using the ‘Cohort’ checklist from CASP for evaluation purposes), and to conduct the review in a manner most amenable to replication.

As with all empirical studies, the present review itself should be considered in light of other reviews (e.g., narrative) that also aim to synthesise the literature in similar and connected areas. It is also acknowledged that the evaluation of the final sample of papers draws an overly critical picture of the current status of research in this area. For example, it would be very difficult for any single study to have scored full marks on all sections of the evaluation criteria. Nevertheless, each of the papers reviewed represents an important contribution to BF/PTG research.

Questions regarding PTG definition have been mentioned, and clarification is a priority, prior to advancing research in understanding BF and PTG development, progression and model-building. Nine specific issues to arise from this heterogeneity of this area of study are given below: (1) the amount of time passed since trauma; (2) demographic variables such as age, gender, and socioeconomic status; (3) medical treatment variations, i.e., seven potential combinations of surgery, radiotherapy and chemotherapy; (4) potential intervening variables that may influence BF/PTG (e.g., emotional support, internal resources such as optimism and resilience); (5) possible confound of current (measured) BF/PTG with prior BF/PTG experiences in response to prior traumatic exposure; (6) the value of using a cut-off score to represent BF/PTG versus the value of a one-item endorsement to represent BF/PTG; (7) indication of illness as representing actual perceived traumatic stress; (8) measurement of BF/PTG as a multi-dimensional versus a general growth construct; and (9) transition between BF to PTG if indeed that occurs.

A number of key conceptual issues related to construct specification can be identified and have yet to be investigated in the reviewed HNC studies. These include the identification of pre- and post-trauma functioning. Determination of whether BF/PTG has occurred in the aftermath of trauma needs to be distinct from an identification of whether it was simply adaptive or superior coping (BF) or the reshaping of self (PTG) that took place. Moreover, identification of BF/PTG through self-report measures might be supplemented with interviews and/or measures for significant others (e.g., family, caregivers). This would enable triangulation of factors and allow for the identification of areas of superior functioning, whether cognitive or behavioural. Qualitative studies would be beneficial in exploring an individual’s history in order to identify any previous trauma, prior coping strategies, resultant PTSD, BF, or PTG that may have occurred, in order to distinguish present psychological coping from past (but possibly ongoing) BF/PTG. An immediate possible way forward in the investigation of BF/PTG would be to conduct between-groups analysis (BF/PTG and non-BF/PTG group) in order to highlight the unique aspects of BF/PTG and the possible benefits that growth may confer. The first step in achieving this would be to assign a value to each measure over which a diagnosis of BF/PTG can be made. The development of the various domains within PTG and cut-offs, might be a focus for future investigations. An example, in health contexts and specifically within cancer, is growth more likely to occur earlier in some domains (e.g., appreciation of life) than in others (e.g., personal strength)? These are important contextual variable that may influence the factors involved in the emergence of BF/PTG in health contexts.

## Conclusion

The five included papers showed a similar pattern of demographic relationships across both constructs of BF and PTG. Similarly, there is overlap with BF/PTG in HNC patients with disease characteristics and psychosocial factors. To enable a fuller understanding of these construct in HNC patients, longitudinal assessment is required using validated measures designed to assess BF & PTG.

## Supplemental Information

10.7717/peerj.256/supp-1 Supplemental Information 1 PRISMA checklist

10.7717/peerj.256/supp-2 Supplemental Information 2 Supplemental Information A: Extraction Search Terms

10.7717/peerj.256/supp-3 Supplemental Information 3 Supplemental Information B: Screening Search Terms

10.7717/peerj.256/supp-4 Supplemental Information 4 Supplemental Information C: NICE Evidence Frameworks

10.7717/peerj.256/supp-5 Supplemental Information 5 Supplemental Information D: CASP questions and quality assessment for the five included studies

10.7717/peerj.256/supp-6 Supplemental Information 6 Supplemental Information E: Description of measures used in the five review studies

## References

[ref-1] Affleck G, Tennen H (1996). Construing benefits from adversity: adaptational significance and dispositional underpinnings. Journal of Personality.

[ref-2] Alter CL, Pelcovitz D, Axelrod A, Goldenberg B, Harris H, Meyers B, Grobois B, Mandel F, Septimus A, Kaplan S (1996). Identification of PTSD in cancer survivors. Psychosomatics.

[ref-3] American Psychiatric Association (2013). Diagnostic and statistical manual of mental disorders.

[ref-4] Andrykowski MA, Cordova MJ, Studts JL, Miller TW (1998). Posttraumatic stress disorder after treatment for breast cancer: prevalence of diagnosis and use of the PTSD Checklist-Civilian Version (PCL-C) as a screening instrument. Journal of Consulting and Clinical Psychology.

[ref-5] Bellizzi KM, Blank TO (2006). Predicting posttraumatic growth in breast cancer survivors. Health Psychology.

[ref-6] Booth A, Fry-Smith A, Petticrew M, Roberts H (2004). Developing a research question. Systematic reviews in the social sciences.

[ref-7] Brennan J (2001). Adjustment to cancer - coping or personal transition?. Psychooncology.

[ref-8] Brennan J, Moynihan C (2004). Cancer in Context: a practical guide to supportive care.

[ref-9] Brunet J, McDonough MH, Hadd V, Crocker PR, Sabiston CM (2010). The posttraumatic growth inventory: an examination of the factor structure and invariance among breast cancer survivors. Psycho-Oncology.

[ref-10] Calhoun LG, Cann A, Tedeschi RG, McMillan J (2000). A correlational test of the relationship between posttraumatic growth, religion, and cognitive processing. Journal of Traumatic Stress.

[ref-11] Calhoun LG, Tedeschi RG, Tedeschi RG, Park CL, Calhoun LG (1998). Posttraumatic growth: future directions. Posttraumatic growth: positive changes in the aftermath of crisis.

[ref-12] Cancer Research UK (2013). http://www.cancerresearchuk.org/cancer-info/cancerstats/.

[ref-13] Carver CS, Antoni MH (2004). Finding benefit in breast cancer during the year after diagnosis predicts better adjustment 5 to 8 years after diagnosis. Health Psychology.

[ref-14] Collins RL, Taylor SE, Skokan LA (1990). A better world or a shattered vision? Changes in life perspectives following victimization. Social Cognition.

[ref-15] Critical Appraisal Skills Programme (2011). http://www.casp-uk.net/.

[ref-16] Doka KJ (2008). Counseling individuals with life-threatening illness.

[ref-17] Eysenck MW (1992). Anxiety: The Cognitive Perspective.

[ref-18] Gallagher-Ross S (2012).

[ref-19] Harrington S, McGurk M, Llewellyn CD (2008). Positive consequences of head and neck cancer: key correlates of finding benefit. Journal of Psychosocial Oncology.

[ref-20] Hartling L, Hamm M, Milne A, Vandermeer B, Santaguida PL, Ansari M, Tsertsvadze A, Hempel S, Shekelle P, Dryden DM (2012). Validity and inter-rater reliability testing of quality assessment instruments (Rep. No. 12-EHC039-EF).

[ref-21] Ho S, Rajandram RK, Chan N, Samman N, McGrath C, Zwahlen RA (2011). The roles of hope and optimism on posttraumatic growth in oral cavity cancer patients. Oral Oncology.

[ref-22] Horney DJ, Smith HE, McGurk M, Weinman J, Herold J, Altman K, Llewellyn CD (2011). Associations between quality of life, coping styles, optimism, and anxiety and depression in pretreatment patients with head and neck cancer. Head & Neck.

[ref-23] Horowitz MJ (1986). Stress response syndrome.

[ref-24] Kunst MJ (2012). Recalled peritraumatic distress in survivors of violent crime: exploring its impact on the relationship between posttraumatic stress disorder symptoms and posttraumatic growth. The Journal of Nervous and Mental Disease.

[ref-25] Larick JG, Graf NM (2012). Battlefield compassion and posttraumatic growth in combat servicepersons. Journal of Social Work Disability Rehabilitation.

[ref-26] Lechner SC, Carver CS, Antoni MH, Weaver KE, Phillips KM (2006). Curvilinear associations between benefit finding and psychosocial adjustment to breast cancer. Journal of Consultant Clinical Psychology.

[ref-27] Leventhal H, Nerenz DR, Steele DF, Baum A, Singer J (1984). Illness representations and coping with health threats. A handbook of psychology and health.

[ref-28] Li Y, Cao F, Cao D, Wang Q, Cui N (2012). Predictors of posttraumatic growth among parents of children undergoing inpatient corrective surgery for congenital disease. Journal of Pediatric Surgery.

[ref-29] Llewellyn CD, Horney DJ, McGurk M, Weinman J, Herold J, Altman K, Smith HE (2011). Assessing the psychological predictors of benefit finding in patients with head and neck cancer. Psycho-Oncology.

[ref-30] Mols F, Vingerhoets AJ, Coebergh JW, van de Poll-Franse LV (2009). Well-being, posttraumatic growth and benefit finding in long-term breast cancer survivors. Psychology & Health.

[ref-31] Morse JM (1997). Responding to threats to integrity of self. Advances in Nursing Science.

[ref-32] Morse JM, Johnson JL, Morse JM, Johnson JL (1991). Towards a theory of illness: the illness constellation model. The illness experience.

[ref-33] Moyer A, Salovey P (1996). Psychosocial sequelae of breast cancer and its treatment. Annals of Behavioral Medicine.

[ref-34] National Institute for Clinical Excellence (2004). Guideline development methods: information for national collaborating centres and guideline developers.

[ref-35] Nunnally JC (1978). Psychometric theory.

[ref-36] Pallant J (2010). SPSS survival manual: A step by step guide to data analysis using SPSS.

[ref-37] Park CL, Ai AL (2006). Meaning-making and growth: new direction for research on survivors of trauma. Journal of Loss and Trauma.

[ref-38] Paton D, Calhoun LG, Tedeschi RG (2006). Posttraumatic growth in disaster and emergency work. Handbook of posttraumatic growth: research and practice.

[ref-39] Petrie KJ, Buick DL, Weinman J, Booth RJ (1999). Positive effects of illness reported by myocardial infarction and breast cancer patients. Journal of Psychosomatic Research.

[ref-40] Petticrew M, Roberts H, Petticrew M, Roberts H (2006). How to appraise the studies: an introduction to assessing study quality. Systematic reviews in the social sciences: a practical guide.

[ref-41] Schriesheim CA, Eisenbach RJ (1995). An exploratory and confirmatory factor-analytic investigation of item wording effects on the obtained factor structures of survey questionnaire measures. Journal of Management.

[ref-42] Schulz R, Williamson GM, Knapp JE, Bookwala J, Lave J, Fello M (1995). The psychological, social and economic impact of illness among patients with recurrent cancer. Journal of Psychosocial Oncology.

[ref-43] Schulz U, Mohamed NE (2004). Turning the tide: benefit finding after cancer surgery. Social Science and Medicine.

[ref-44] Sears SR, Stanton AL, Danoff-Burg S (2003). The yellow brick road and the emerald city: benefit finding, positive reappraisal coping and posttraumatic growth in women with early-stage breast cancer. Health Psychology.

[ref-45] Seery MD (2011). Resilience: a silver lining to experiencing adverse life events?. Psychological Science.

[ref-46] Stanton AL, Bower JE, Low CA, Calhoun LG, Tedeschi RG (2006). Posttraumatic growth after cancer. Handbook of posttraumatic growth: research and practice.

[ref-47] Stoll C, Schelling G, Goetz AE, Kilger E, Bayer A, Kapfhammer HP, Rothenhausler HB, Kreuzer E, Reichart B, Peter K (2000). health-related quality of life and post-traumatic stress disorder in patients after cardiac surgery and intensive care treatment. Journal of Thoracic and Cardiovascular Surgery.

[ref-48] Sumalla EC, Ochoa C, Blanco I (2009). Posttraumatic growth in cancer: reality or illusion?. Clinical Psychology Review.

[ref-49] Tedeschi RG, Calhoun LG (1996). The posttraumatic growth inventory: measuring the positive legacy of trauma. Journal of Trauma Stress.

[ref-50] Tennen H, Affleck G, Snyder CR, Lopez SJ (2002). Benefit-finding and benefit-reminding. The handbook of positive psychology.

[ref-51] The Cochrane Collaboration (1999). http://hiru.mcmaster.ca/cochrane/cochrane/hbook.htm.

[ref-52] Thomson ResearchSoft (2000).

[ref-53] Timberlake N, Klinger L, Smith P, Venn G, Treasure T, Harrison M, Newman SP (1997). Incidence and patterns of depression following coronary artery bypass graft surgery. Journal of Psychosomatic Research.

[ref-54] Tomich PL, Helgeson VS (2004). Is finding something good in the bad always good? Benefit finding among women with breast cancer. Health Psychology.

[ref-55] Wilson Van Voorhis CR, Morgan BL (2013). Understanding power and rules of thumb for determining sample sizes. Tutorials in Quatitative Methods for Psychology.

[ref-56] Zoellner T, Maercker A (2006). Posttraumatic growth in clinical psychology – A critical review and introduction of a two component model. Clinical Psychology Review.

