# Supplementary material for: Existence of benefit finding and posttraumatic growth in people treated for head and neck cancer: a systematic review
Source: PeerJ. 2014 Feb 11;2:e256. doi: 10.7717/peerj.256 (PMC3933269; doi:10.7717/peerj.256)
Supplement: Supplemental Information 2 [file peerj-02-256-s002.doc]

Supplemental Information A: Extraction Search Terms

1. Neoplasm$

2. Tumo?r$

3. Cancer$

4. Benign Neoplasm$

5. Growth$

6. Oncology

7. Melanoma$

8. Malignant Melanoma$

9. Squamous Cell

10. Squamous Cell Neoplasm$

11. Squamous Cell Cancer$

12. Squamous Cell Tumo?r$

13. #1 or #2 or #3 or #4 or #5 or #6 or #7 or #8 or #9 or #10 or #11 or #12

14. Stress-Related Growth

15. Stress Related Growth

16. Posttraumatic Growth

17. Post Traumatic Growth

18. Post-Traumatic Growth

19. Existential Growth

20. Thriving

21. Transformational Coping

22. Positive Change

23. Perceived benefit$

24. Perception of benefit$

25. Positive adjustment

26. Positive adaptation

27. Adversarial growth

28. Benefit Finding

29. Changes in Outlook Questionnaire

30. CiOQ

31. COPE Scale

32. Perceived Benefit Scale

33. PBS

34. Posttraumatic Growth Inventory

35. PTGI

36. Modified Posttraumatic Growth Inventory

37. PTGI-M

38. Professioanl Quality of Life Measure

39. ProQoL

40. Stress-Realted Growth Scale

41. SRGS

42. Revised Stress-Realted Growth Scale

43. SRGS-R

44. RSRGS

45. Short Form Stress-Realted Growth Scale

46. Stress-Realted Growth Scale Short Form

47. SRGS-S

48. Thriving Scale

49. TS

50. Ilness Cognition Questionnaire

51. ICQ

52. Silver Lining Questionnaire

53. SLQ

54. #14 or #15 or #16 or #17 or #18 or #19 or #20 or #21 or #22 or #23 or #24 or #25 or #26 or #27 or #28 or #29 or #30 or #31 or #32 or #33 or #34 or #35 or #36 or #37 or #38 or #39 or #40 or #41 or #42 or #43 or #44 or #45 or #46 or #47 or #48 or #49 or #50 or #51 or #52 or #53

55. #13 and #54
