# Supplementary material for: Existence of benefit finding and posttraumatic growth in people treated for head and neck cancer: a systematic review
Source: PeerJ. 2014 Feb 11;2:e256. doi: 10.7717/peerj.256 (PMC3933269; doi:10.7717/peerj.256)
Supplement: Supplemental Information 3 [file peerj-02-256-s003.doc]

Supplemental Information B: Screening Search Terms

1. Craniofacial

2. Ear

3. Esophageal

4. Esophagus

5. Eyelid

6. Facial

7. Floor of Mouth

8. Gingival

9. Glottis

10. Head and Neck

11. Laryngeal

12. Larynx

13. Leukoplakia Oral

14. Lip

15. Mandible

16. Maxilla

17. Maxillofacial

18. Mouth

19. Nasal

20. Nose

21. Oral

22. Oral Cavity

23. Otorhinolarngology

24. Otorhinolaryngologic

25. Palatal

26. Parathyroid

27. Parotid

28. Pharyngeal

29. Pharynx

30. Salivary Gland$

31. Soft Palate

32. Supraglottis

33. Throat

34. Thyroid

35. Thyroid Nodule

36. Tongue

37. Tonsil

38. Tracheal

39. Transglottis

40. Vocal Cords

Not

1. Acoustic Neuroma
2. Brain

Skin
