# Supplementary material for: Existence of benefit finding and posttraumatic growth in people treated for head and neck cancer: a systematic review
Source: PeerJ. 2014 Feb 11;2:e256. doi: 10.7717/peerj.256 (PMC3933269; doi:10.7717/peerj.256)
Supplement: Supplemental Information 4 [file peerj-02-256-s004.doc]

Supplemental Information C: NICE Evidence Frameworks

| **Evidence Category** | **Source** |
| --- | --- |
| Ia | Systematic review and meta-analysis or randomised controlled trials (RCTs) |
| Ib | At least one RCT |
| IIa | At least one well-designed controlled study without randomisation |
| IIb | At least one other type of well-designed quasi-experimental study |
| III | Well-designed non-experimental descriptive studies, such as comparative studies or case studies |
| IV | Expert committee reports or opinions and/or clinical experience or respected authorities |
