# Supplementary material for: Existence of benefit finding and posttraumatic growth in people treated for head and neck cancer: a systematic review
Source: PeerJ. 2014 Feb 11;2:e256. doi: 10.7717/peerj.256 (PMC3933269; doi:10.7717/peerj.256)
Supplement: Supplemental Information 5 [file peerj-02-256-s005.doc]

Supplemental Information D: CASP questions and quality assessment for the five included studies

|  | Harrington, S., McGurk, M. & Llewellyn, C.D. (2008) | Llewellyn, C.D., Horney, D.J., McGurk, M., Weinman, J., Herold, J., Altman, K. & Smith, H.E. (2011) | Ho, S.M.Y., Rajandram, R.K, Chan, N., Samman, N., McGrath, C. & Zwahlen, R.A. (2011) | Harding, S. & Moss, T.P. (In Submission a) | Harding, S. & Moss, T.P. (In Submission b) |
| --- | --- | --- | --- | --- | --- |
| **1.** Did the study address a clearly focused issue? | Yes | Yes | Yes | Yes | Yes |
| **2.** Did the authors use an appropriate method to answer the question? | Yes | Yes | Yes | Yes | Yes |
| **3.** Was the cohort recruited in an acceptable way? | Yes. Postal survey (55% return rate) | Yes. At Clinic visits | Yes. At Clinic visits | Yes. Postal survey  (53% return rate) | Yes. Postal survey (87% return rate at second time point) |
| **4.** Was the exposure accurately measured to minimise bias? | Yes | Yes | Yes | Yes | Yes |
| **5.** Was the outcome accurately measured to minimise bias? | Yes | Yes | Yes | Yes | Yes |
| **6a.** Have the authors identified all important confounding factors? | Yes | No. Additional journal articles were required to source information on confounding variables | Yes | Yes | Yes |
| **6b.** Have they taken account of the confounding factors in the design and/or analysis? | Yes – Pearson’s correlation, Stepwise linear regression, Independent Sample t-test | Yes - Independent Sample t-test, Wilcoxen Signed Rank Test, Spearman correlation, Principle Component Analysis, Multivariate linear regression | Yes – Pearson correlation, Analysis of Variance, Independent Sample t-test with Bonferroni Correction, Regression | Yes – Mann-Whitney U Tests, Spearman correlation, Step wise linear regression | Yes – Mann-Whitney U Test, Spearman Correlation, Analysis of Variance, Mixed Linear Effects Modelling |
| **7a.** Was the follow up of subjects complete enough? | No. Only one time point for each participant, even though this represented a range of 6 to 120 months post treatment | No. Only 66% of patients completed the second time point (6mths) | No. Only one time point for each participant, but no numerical data is provide about length of time from treatment other than it is greater than 6mths | No. Only one time point for each participant, even though this represented a range of 3 to 76 months post treatment | Yes. 142 (87%) complete data sets were available for analysis at two time points. Range for time at time point 1 was between 3 & 113 months (Mean 32) and for time point 2 was between 15 & 125 months (Mean 45) |
| **7b.** Was the follow up of subjects long enough? | No. The sample size with all the potential confounders makes the data insufficiently robust to be long enough | No. Follow up only at 6mths | No. The sample size with all the potential confounders makes the data insufficiently robust to be long enough | No. The sample size with all the potential confounders makes the data insufficiently robust to be long enough | This is an improvement on previous work by increasing the follow up time and including a greater sample size to account for additional variables |
| **8.** What are the results? | Discussed in the body of the manuscript | Discussed in the body of the manuscript | Discussed in the body of the manuscript | Discussed in the body of the manuscript | Discussed in the body of the manuscript |
| **9.** How precise are the results? | The measures used with the study all demonstrate a good level of reliability and validity. Appropriate statistics were used. | The Measures used in this study have demonstrated appropriate levels of reliability and validity, except for the two questions selected from the EORTC. Analysis was appropriate for the data, but Type II errors may be present due to the small sample size and the large statistical demand. | The authors of this paper are responsible for the development of the Chinese versions of the scales used. It is not clear if the data presented within the paper were part of this validation work. Analysis was appropriate, although sample size was small. | The measures used with the study all demonstrate a good level of reliability and validity. Appropriate statistics were used. | The measures used within the study all demonstrate a good level of reliability and validity. Appropriate statistics were used and sufficient power due to sample size is present. |
| **10.** Do you believe the results | Can't tell. Low statistical power given the number of factors. | Yes | Can't tell. Low statistical power given the number of factors. | Yes | Yes |
| **11.** Can the results be applied to the local population? | No. Comparison with other studies or normative values has not been made with any of the questionnaires | Can't tell. No attempts made to compare presented data with normative values available from SF-12, or from disease populations using HADS | No. This is a Chinese population and it is possible that there are ethnic variations between the study population and a western population | Yes. Made possible by comparison with normative data from the SF-12 and disease comparison with UoW | Yes. Made possible by comparison with normative data from the SF-12 and disease comparison with UoW. Additionally the sample size makes it likely that reliable cross population comparisons can be made |
| **12.** Do the results of this study fit other available evidence? | Small sample size and this being the first study of its type in this patient cohort makes it difficult to comment | This is only the second time BFS has been used in this disease group and the first occasion was from the same research group | Small sample size. The PTGI has not been used in this disease cohort before | The SLQ has not been used in this disease cohort before | This is only the second time that the SLQ measure has been used in this disease group and the first occasion was from the same research group. The results obtained in this analysis support the previous findings |
| **Assessment of Quality within NICE (2004) guidelines** | Level IIb | Level IIb | Level IIb | Level IIb | Level IIb |
| **Assessment of Quality Guided by the CASP Cohort Checklist** | Low | Medium | Low | Medium | Medium |
