# Supplementary material for: Existence of benefit finding and posttraumatic growth in people treated for head and neck cancer: a systematic review
Source: PeerJ. 2014 Feb 11;2:e256. doi: 10.7717/peerj.256 (PMC3933269; doi:10.7717/peerj.256)
Supplement: Supplemental Information 6 [file peerj-02-256-s006.doc]

Supplemental Information E: Description of measures used in the five review studies

| **Study using the measure** | **Measure** | **Measure characteristics** |
| --- | --- | --- |
| Harrington, S., McGurk, M. & Llewellyn, C.D. (2008)  Llewellyn, C.D., Horney, D.J., McGurk, M., Weinman, J., Herold, J., Altman, K. & Smith, H.E. (2011) | Benefit finding scale (BFS), | This 17-item scale (Antoni et al., 2001) was originally developed for use with patients with breast cancer and was adapted by us for use with HNC patients by using the stem, ‘Having head and neck cancer. . .’ followed by potential benefits from the experience. The items focused on potential benefits ranging from family and social relationships, life priorities, sense of spirituality and ability to accept the circumstances. Responses were made on a five-item Likert scale (from strongly disagree to strongly agree). Six items were negatively worded and reverse coded prior to analysis. Total scores ranged from 17 to 85, with higher scores indicating higher levels of BF. Internal consistency in this sample was a = 0.76 (pre-treatment) and a = 0.81 (post-treatment). |
| Llewellyn, C.D., Horney, D.J., McGurk, M., Weinman, J., Herold, J., Altman, K. & Smith, H.E. (2011) | Brief COPE | The Brief COPE is a validated multidimensional coping inventory, which assesses situational coping (Carver, 1997). The short-form version of the COPE assesses coping strategies on 14 conceptually different subscales, which comprise self-distraction (a = 0.41), active coping (a = 0.47), denial (a = 0.70), substance use (a = 0.91), use of emotional support (a = 0.68), use of instrumental support (a = 0.64), behavioural disengagement (a = 0.38), venting (a = 0.51), positive reframing (a = 0.49), planning (a = 0.44), humour (a = 0.92), acceptance (a = 0.74), religion (a = 0.92) and self-blame (a = 0.68). Each subscale has two items. Scores ranged from 1 (‘don’t do this at all’) to 4 (‘do this a lot’), with higher scores indicating greater use of a particular coping style. |
| Ho, S.M.Y., Rajandram, R.K, Chan, N., Samman, N., McGrath, C. & Zwahlen, R.A. (2011) | Chinese Posttraumatic Growth Inventory (PTGI), | The PTGI and its Chinese version validated by Ho and colleagues (2004) is a 21-item scale to assess posttraumatic growth (Tedeschi & Calhoun, 1996). The PTGI includes the interpersonal and intrapersonal dimensions of growth. Items were rated on a 6-point scale (0 = not at all, 5 = very much indeed). The internal reliability alpha value for the scale was 0.85 in the current study. |
| Ho, S.M.Y., Rajandram, R.K, Chan, N., Samman, N., McGrath, C. & Zwahlen, R.A. (2011) | Chinese Version of Hope scale (HS), | The Hope Scale and its Chinese version by Ho et al. (2010)was used to assess patients levels of hope (Snyder et al., 2001). The scale contains four Agency and four Pathways items, which were mixed with four distracter items in administration. Each item is rated on a 4-point Likert scale (1 = strongly disagree, 4 = strongly agree) in which a high score indicates a higher level of pathways and agency component. Internal reliability alpha values were calculated at 0.76, 0.70, and 0.81, respectively for the pathways subscale, agency subscale and total hope scale. |
| Harrington, S., McGurk, M. & Llewellyn, C.D. (2008)  Llewellyn, C.D., Horney, D.J., McGurk, M., Weinman, J., Herold, J., Altman, K. & Smith, H.E. (2011) | Hospital Anxiety and Distress Scale (HADS), | Hospital Anxiety and Depression Scale (HADS) (Zigmond & Snaith, 1983). The HADS is a validated 14-item self-assessment scale with scores ranging from 0 to 21, with higher scores indicating greater anxiety and depression. Scores between 8 and 10 indicate a possible clinical disorder and scores of 11 or more probable psychiatric morbidity. The internal consistency in this sample was a = 0.84 for anxiety and a = 0.85 for depression (Zigmond & Snaith, 1983). |
| Harrington, S., McGurk, M. & Llewellyn, C.D. (2008)  Llewellyn, C.D., Horney, D.J., McGurk, M., Weinman, J., Herold, J., Altman, K. & Smith, H.E. (2011) | Life Orientation Test - Revised (LOT-R), | The LOT-R is used to assess the patients’ level of optimism (Scheier & Carver, 1985). The scale comprises six items, in which three positive- and three negative-worded measure positive and negative outcome expectancies respectively. A 4-point Likert scale (1 = strongly disagree, 4 = strongly agree) is used to rate the patients response. The internal reliability alpha (a = 0.66) value for optimism in the current study was moderate but acceptable. |
| Ho, S.M.Y., Rajandram, R.K, Chan, N., Samman, N., McGrath, C. & Zwahlen, R.A. (2011) | Chinese Version of Life Orientation Test - Revised (LOT-R), | The LOT-R is used to assess the patients’ level of optimism (Scheier & Carver, 1985). The scale comprises six items, in which three positive- and three negative-worded measure positive and negative outcome expectancies respectively. A 4-point Likert scale (1 = strongly disagree, 4 = strongly agree) is used to rate the patients response. The internal reliability alpha (a = 0.66) value for optimism in the current study was moderate but acceptable (Lai et al., 1998). |
| Llewellyn, C.D., Horney, D.J., McGurk, M., Weinman, J., Herold, J., Altman, K. & Smith, H.E. (2011)  Harding, S & Moss, T.P. (In Submission a)  Harding, S. & Moss, T.P. (In Submission b) | Medical Outcomes Short Form 12 (SF-12), | The SF-12 is a multipurpose self-administered short form questionnaire with 12 items, all selected from the SF-36 Health Survey (Ware, Jr. et al., 1996). It is a generic QoL measure, as opposed to one that targets a specific age, disease, or treatment group. It has proven to be useful in measuring outcomes in clinical trials, is well validated and brief enough to minimise response burden (Melville et al., 2003; Stoll et al., 2000). Results for each patient are expressed in terms of two meta-scores; Physical Component Summary (PQoL) and Mental Component Summary (MQoL). The PQoL and MQoL scores have a range of 0 to 100. Scores greater than 50 represent above average HRQoL status compared with age and gender matched normative values (Ware, Jr. et al., 1996), whereas a score of 40 indicates a function at a level lower than 84% of the population (one standard deviation), and people with a score less than 30 function at a level lower than approximately 98% of the population (two standard deviations).The SF-12 has demonstrated internal consistencies of α = 0.71 – 0.90 for all of the sub-scales including the PQoL and MQoL and test-retest of between r = 0.71 and 0.84 (Ware, Jr. et al., 1996). |
| Harding, S. & and Moss, T.P. (In Submission a)  Harding, S. & Moss, T.P. (In Submission b) | Silver Lining Questionnaire (SLQ), | The SLQ measures the extent to which people believe their illness has had a positive benefit despite the negative consequences of being ill (Sodergren & Hyland, 2000). Research suggests that this positive interpretation reflects personal growth (i.e. PTG). The SLQ is a 38-item scale was selected for its psychometric properties and its reported ability to measure PTG. The SLQ measures 10 facets of positivity with illness: restructuring of life; reappraisal of life; spiritual gains; self-improvement; self-awareness; skills and new pursuits; sensitivity to emotions; confrontation of current concerns; improved interpersonal relationships; and positive consequences for others. Cronbach’s alpha is 0.93 and retest reliability is r = 0.90 (Sodergren et al., 2002). |
| Harding, S. & Moss, T.P. (In Submission a)  Harding, S. & Moss, T.P. (In Submission b) | University of Washington Head and Neck Caner Quality of Life (UoW), | The UoW is a broad measure of HRQoL for HNC with good patient acceptability, practicality, validity, reliability, and responsiveness (Hassan & Weymuller, Jr., 1993). Not only does the UoW measure HRQoL items, it can predict clinical factors such as length of stay in hospital and loss of chewing and swallowing function (Rogers et al., 2001), and identify discrete patient groups (Rogers et al., 2000). The UoW quality of life questionnaire version four was used and covers 12 domains: pain, appearance, activity, recreation, swallowing, chewing, speech, shoulder function, taste, saliva, mood and anxiety. The UoW has been validated by comparison to the Karnofsky scale and Sickness Impact Profile demonstrating an average criterion validity of 0.85 (Hassan & Weymuller, Jr., 1993). It has also been found to have internal consistency between α = 0.80 and 0.79 and Test-retest of r = 0.91 (Rogers et al., 2002). |
